# Characterization of mechanical stiffness using additive manufacturing and finite element analysis: potential tool for bone health assessment

**DOI:** 10.1186/s41205-023-00197-5

**Published:** 2023-11-18

**Authors:** Sriharsha Marupudi, Qian Cao, Ravi Samala, Nicholas Petrick

**Affiliations:** Division of Imaging, Diagnostics, and Software Reliability, Office of Science and Engineering Labs, U.S. Food and Drug Administration, Silver Spring, MD USA

**Keywords:** Trabecular bone, Additive manufacturing, Micro–finite element analysis, Compression testing, Micro-computed tomography, Bone microstructure

## Abstract

**Background:**

Bone health and fracture risk are known to be correlated with stiffness. Both micro-finite element analysis (μFEA) and mechanical testing of additive manufactured phantoms are useful approaches for estimating mechanical properties of trabecular bone-like structures. However, it is unclear if measurements from the two approaches are consistent. The purpose of this work is to evaluate the agreement between stiffness measurements obtained from mechanical testing of additive manufactured trabecular bone phantoms and μFEA modeling. Agreement between the two methods would suggest 3D printing is a viable method for validation of μFEA modeling.

**Methods:**

A set of 20 lumbar vertebrae regions of interests were segmented and the corresponding trabecular bone phantoms were produced using selective laser sintering. The phantoms were mechanically tested in uniaxial compression to derive their stiffness values. The stiffness values were also derived from in silico simulation, where linear elastic μFEA was applied to simulate the same compression and boundary conditions. Bland-Altman analysis was used to evaluate agreement between the mechanical testing and μFEA simulation values. Additionally, we evaluated the fidelity of the 3D printed phantoms as well as the repeatability of the 3D printing and mechanical testing process.

**Results:**

We observed good agreement between the mechanically tested stiffness and μFEA stiffness, with *R*^*2*^ of 0.84 and normalized root mean square deviation of 8.1%. We demonstrate that the overall trabecular bone structures are printed in high fidelity (Dice score of 0.97 (95% CI, [0.96,0.98]) and that mechanical testing is repeatable (coefficient of variation less than 5% for stiffness values from testing of duplicated phantoms). However, we noticed some defects in the resin microstructure of the 3D printed phantoms, which may account for the discrepancy between the stiffness values from simulation and mechanical testing.

**Conclusion:**

Overall, the level of agreement achieved between the mechanical stiffness and μFEA indicates that our μFEA methods may be acceptable for assessing bone mechanics of complex trabecular structures as part of an analysis of overall bone health.

## Introduction

Trabecular bone is a honeycomb-like network of spongy and porous material present in vertebrae and long bones [1]. It is the primary load bearing structure in vertebral bodies and has stiffness values ranging from 1.6 × 10^4^ N/mm – 4.0 × 10^11^ N/mm [2, 3]. Bone health is dependent on trabecular bone microstructure which can be modeled as a collection of rods (e.g., elongated, cylindrical regions) and plates (i.e., extended, flatter regions) [4, 5]. Plates are orientated along the anatomic load bearing axis, while rods connect and stabilize plates. The microarchitecture of trabecular rods and plates can be characterized by volume, orientation, and number of structures. Skeletal diseases such as osteoporosis and osteopenia occur due to changes in microstructure and material properties of bone [6]. Osteoporosis is a metabolic disease characterized by low bone mineral density (BMD) that reduces bone strength and increases fracture risk [7]. Osteopenia designates a decrease in BMD but not low enough to be considered osteoporotic [8]. Assessment of bone strength and fracture risk is important for staging and monitoring of osteoporosis. These disorders are currently diagnosed using Dual Energy X-ray Absorptiometry (DEXA), but DEXA scans do not fully capture the 3D bone microstructure and morphology [9].

Bone microstructure can be quantitively assessed ex vivo using micro computed tomography (μCT) [10]. Finite element analysis (FEA) and micro-finite element analysis (μFEA) can be used to estimate the mechanical properties of trabecular bone in silico. In these simulations, a mesh representing trabecular bone microstructure is broken into small tetrahedral volume elements. A boundary condition can be applied to each element (i.e., compression, stationary), and the deformation for all elements in the system can be solved using differential equations in continuum mechanics. These methods have been applied to μCT, multidetector computed tomography (MDCT), and high-resolution peripheral quantitative computed tomography (HR-pQCT) images of trabecular bone to evaluate stiffness and strength [11, 12]. However, validation studies on the accuracy and precision of these μFEA models are limited in the literature [13, 14]. In these studies, mechanical tests were conducted on trabecular bone samples that were scanned by HR-pQCT and μCT systems. These 3D scans were then used to build μFEA models to compute stiffness estimates. The validation studies show that μFEA is a robust method for predicting mechanical properties of bone. In vitro mechanical testing of trabecular bone tissue is used to validate μFEA simulation results [13, 15–18].

Advancements in additive manufacturing allow for the reproduction of bone microarchitecture in 3D printed phantoms [19]. These phantoms can potentially be used to evaluate trabecular bone mechanical properties in lieu of real tissue. There are many previous studies on additive manufacturing and mechanical testing of trabecular bone. They include: comparing the mechanical properties of 3D printing trabecular bone phantoms with different microarchitectures [20], compression testing of trabecular bone phantoms at various scaling factors [21], and varying the 3D printing parameters to observe changes in the mechanical properties of the phantoms [22]. These studies indicate that 3D printed trabecular bone phantoms are able to predict the mechanical properties of trabecular bone. Similar to our approach in this work, the 3D printed phantoms mentioned here are scaled up and do not reflect trabecular bone structures at their native scale.

Printing technologies commonly used for 3D printing trabecular bone structures include stereolithography (SLA) and selective laser sintering (SLS) [23]. SLA printers use an ultraviolet laser beam to cure the resin material and create a solid layer [24]. This process is performed several times to build the model layer by layer. Supports are inserted into the model for structural integrity. The final step involves curing the printed model until the resin completely hardens. In contrast, SLS printers generate solid models through lasers fusing material powders. The powder is melted by the laser layer by layer until the final structure is formed. Powder that is not fused acts as a support structure for the model [25].

There are several benefits to evaluating the mechanical properties of trabecular bone through additive manufacturing. First, 3D printed phantoms are easier to acquire than cadaveric trabecular bone samples. Additionally, machining trabecular bone phantoms for mechanical testing can be physically challenging. Coring of trabecular bone phantoms requires specialized equipment such as diamond drills and can damage structures in the cored specimen [26]. 3D printed phantoms also do not require temperature control or need to be immersed in saline solution to maintain consistent mechanical properties [21, 27]. In contrast, the mechanical properties of cadaveric samples will change each time they are removed from the saline solution or thawed. Multiple 3D printed phantoms can be generated for mechanical testing, each with a unique bone microarchitecture. Additionally, boundary conditions can be consistently set and reproduced for every test. In contrast, cadaveric samples are can only be tested once and difficult to fix during mechanical testing and thus, may not have well defined boundary conditions.

There are several challenges for additive manufacturing trabecular bone. The scale of the 3D printed structures is limited by printer resolution. Currently, with some exceptions, it is not possible to manufacture structures at the native scale of trabecular bone using commercially available 3D printers [28]. Thus, structures must be scaled up to retain fidelity to the original model and ameliorate printing defects. The material properties of the 3D printing substrate are not equivalent to real bone. Bone is a heterogeneous, anisotropic, non-linear material and has an elastic modulus dependent on anatomic site and orientation of the microstructure [1]. If a phantom is loaded off axis during mechanical testing, there can be a significant difference in mechanical properties [29]. Additive manufactured substrates are generally isotropic with a uniform elastic modulus within the phantom. Structures such as canaliculi and the bone nanostructure are not replicated in the 3D printed phantom.

There are also several disadvantages to using 3D printing to evaluate trabecular bone mechanical properties. 3D printed phantoms may not be an accurate representation of the in silico model. Structures can be geometrically inaccurate resulting in errors in the μFEA stiffness estimations. There may also be 3D printing defects in the phantom such as porosity, unsintered powder, curled edges, or missing structures [30, 31]. Additionally, 3D printing may introduce anisotropy into the phantom and this anisotropy can aggregate as the phantom is built layer by layer. Last, the experimental boundary conditions must be carefully controlled during mechanical testing, and properly modeled in silico to yield accurate stiffness results.

For evaluating our μFEA model: we performed testing to demonstrate that given accurate 3D printing, quantities such as stiffness are linear to scaling [32]. Elastic modulus can be assumed to be isotropic locally in a small patch of the bone. This is consistent with assumptions in continuous mechanics [33]. Therefore, a first order approximation of stiffness via linear elasticity is possible. Additionally, we limit our analysis to assessment of relative stiffness values instead of absolute stiffness values by calibrating our μFEA model to a simple phantom that can be 3D printed and mechanically tested accurately.

In this work, we evaluate the agreement between stiffness results derived from μFEA simulations and stiffness results from mechanical testing of trabecular bone phantoms. We first assess the ability of existing 3D printing technology to produce trabecular bone phantoms that are both morphologically accurate and mechanically stable. Then, by mechanically testing these phantoms, we can provide independent validation for our μFEA simulation. The phantoms are produced from μCT image data of multiple lumbar vertebrae samples. The bone phantoms are produced at a larger scale than their native resolution to improve printing fidelity. Prior studies have only 3D printed phantoms based on image data from one bone sample [20, 21]. In those studies, the bone sample was varied in scaling, material, or morphology, and then compression tested [20–22]. Incorporating several different bone samples with variable morphologies provides for a more robust evaluation of μFEA modeling. We also evaluated the repeatability of 3D printing by mechanically testing multiple copies of the same phantom. The bone phantoms in this study range in bone health such that they are comprised of samples that are healthy and with varying degrees of osteoporosis. The samples also have diverse microarchitectures, some samples have more rod-like trabecular structures and others have more plate-like structures.

## Materials and methods

### Dataset and image processing

Seventy-six lumbar vertebral bodies (L1-L5) were imaged with μCT (micro-CT 100, Scanco Medical, Basserdorf, Switzerland). The patients were male and female (ages 44-98) with varying levels of bone health. The scans were conducted in air with a 70 kVp X-ray source and a voxel size of 51.3 μm. Several vertebrae in the dataset were osteoporotic and osteopenic with cavities, deteriorated bone structure, and fractures. Other imaging artifacts were due to sample preparation and image acquisition. Sample preparation also led to many of the samples having air bubbles in place of bone marrow. As a result of the imaging acquisition protocol some images contained metal artifacts due to beam hardening and scattering from pedicle screws inserted in the vertebrae.

Three-dimensional regions of interests (ROIs) were extracted from the images using 3D Slicer [34]. Coordinates corresponding to the centers of the ROIs were manually chosen to avoid including cortical bone and imaging artifacts in the ROI. Samples that were more osteoporotic had either limited trabeculae and/or large cavities. Additionally, the ROIs were rotated to align with the principal axis of the trabecular bone structures. This is needed to achieve consistent measurement of stiffness due to the anisotropy of trabecular bone structures [35]. Ten ROIs were extracted from each bone image. Extracted ROIs had a volume of approximately 3.6 cm^3^, and the volume was reduced to approximately 1 cm^3^ to remove imaging artifacts from the edge of the ROI.

The selected ROIs were then, binarized to differentiate trabecular bone voxels from cortical bone voxels. Gaussian blur, anisotropic diffusion, and Otsu thresholding were applied to reduce noise and segment the images. Each of the slices was inspected along the primary axes to correct for segmentation error.

### Microstructure metrics

The ROIs were analyzed using BoneJ, a Fiji plugin for microstructure analysis of skeletal biology [36–38]. All images were converted to 8-bit NRRD images for analysis in BoneJ. To quantitatively assess the trabecular bone microstructure, the following microstructure metrics were selected: thickness, spacing, anisotropy, bone volume fraction (BV/TV), ellipsoid factor (EF), and connectivity density. These metrics are commonly reported in the literature for quantitative characterization of trabecular bone morphology [39].

Thickness is the mean thickness of the trabecular structures, while spacing is the mean distance between trabecular structures [38]. BV/TV is defined as the ratio of mineralized bone in the sample to the total volume of the bone [39]. Anisotropy quantifies trabecular bone’s directionality with the degree of anisotropy representing the microstructure’s orientation. A degree of anisotropy closer to 0 indicates that the bone is more isotropic, while values closer to 1 indicate that the bone is more anisotropic [40]. Connectivity determines the number of connected structures in a sample. The connected structures are representative of trabeculae in a trabecular network [36]. Connectivity is generally presented as connectivity density, the connectivity divided by the total volume of the image [39]. Lastly, EF quantifies the rod and plate geometry of the trabecular microstructure [41]. EF is evaluated on a scale of − 1 to + 1, with − 1 corresponding to an oblate plate-like geometry, and + 1 corresponding to a prolate rod-like geometry. The trabecular microstructure transitions from plate-like to rod-like geometry as bone strength is lost [42].

From the segmented ROIs, 20 were chosen to be printed using an SLS EOS P396 printer with Nylon 12 material (Elastic modulus of 1650 MPa) and layer resolution of 120 μm. The ROIs were selected based on microstructure metrics and trabecular morphology to have a diverse dataset. BV/TV values were the largest factor in selecting ROIs as this metric directly is correlated with the stiffness of trabecular bone [43].

### Cropping trabecular bone ROIs

The ROIs were cropped from the original size to remove residual cortical bone from the edge of the image, mitigate the effect of imaging artifacts, and reduce the number of trabecular structures for 3D printing. Cortical bone was present on the edges of ROIs along with pedicle screw artifacts, cavities, and fractures. The ROIs were cropped rather than pruned to conserve the connectivity of the trabecular structures. An ROI with many complex trabecular structures is challenging to print without obvious defects or geometric errors. However, if the ROIs are cropped such that too few microstructures are included, the ROIs lose homogeneity and resemblance to real bone.

We calculated the difference in microstructure metrics by comparing the cropped ROIs to the original ROIs to evaluate how the metrics changed with varying volume size. The final volume was chosen as the smallest ROI with microstructure metrics similar to the original ROI. Microstructure metrics such as thickness, spacing, and anisotropy had a modest difference of approximately 8-14% compared to the original ROI. This is below the 13-12 intravertebral variation observed in trabecular microstructure [44–46]. However, BV/TV and connectivity density had a difference of 27-28%, which is greater than the 22% difference generally observed in trabecular bone These metrics are sensitive to changes in the number of structures in the sample. Based on this result we believe the selected ROIs contain a reasonable number of trabecular structures for our analysis.

**Fig. 1:**
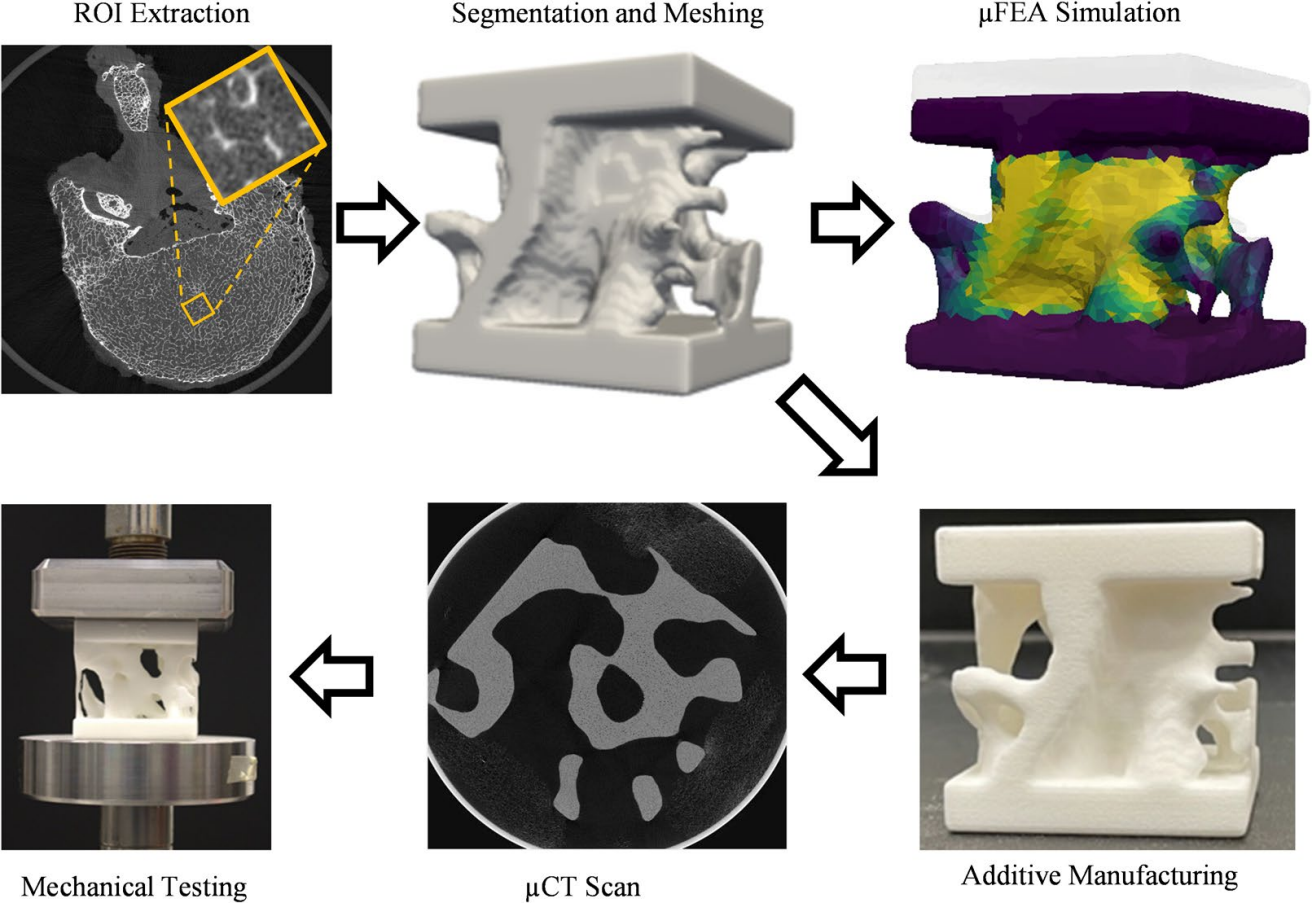
Summary of workflow including, ROI extraction, segmentation, meshing, μFEA stiffness estimation, additive manufacturing trabecular bone phantoms, μCT characterization, and mechanical testing

Visualizations of the ROIs were generated to determine whether the trabecular structures still resembled real bone. A volume of (2.5 × 2.5 × 2.5) mm^3^ was chosen for 3D printing and scaled to have dimensions of (50x50x50) mm^3^. Due to the resolution limit of the SLS printer, phantoms cannot be printed at the native resolution of trabecular bone. To print the phantoms with geometric accuracy the ROIs must be scaled up. The final trabecular bone phantoms are a reasonable size for 3D printing and mechanical testing (Fig. 1).

## Finite element analysis and mesh convergence study

### Finite element model and analysis

The μFEA model in this work uses voxelized models of trabecular bone and converts them into tetrahedral meshes to simulate linear trabecular bone mechanics.

**Fig. 2:**
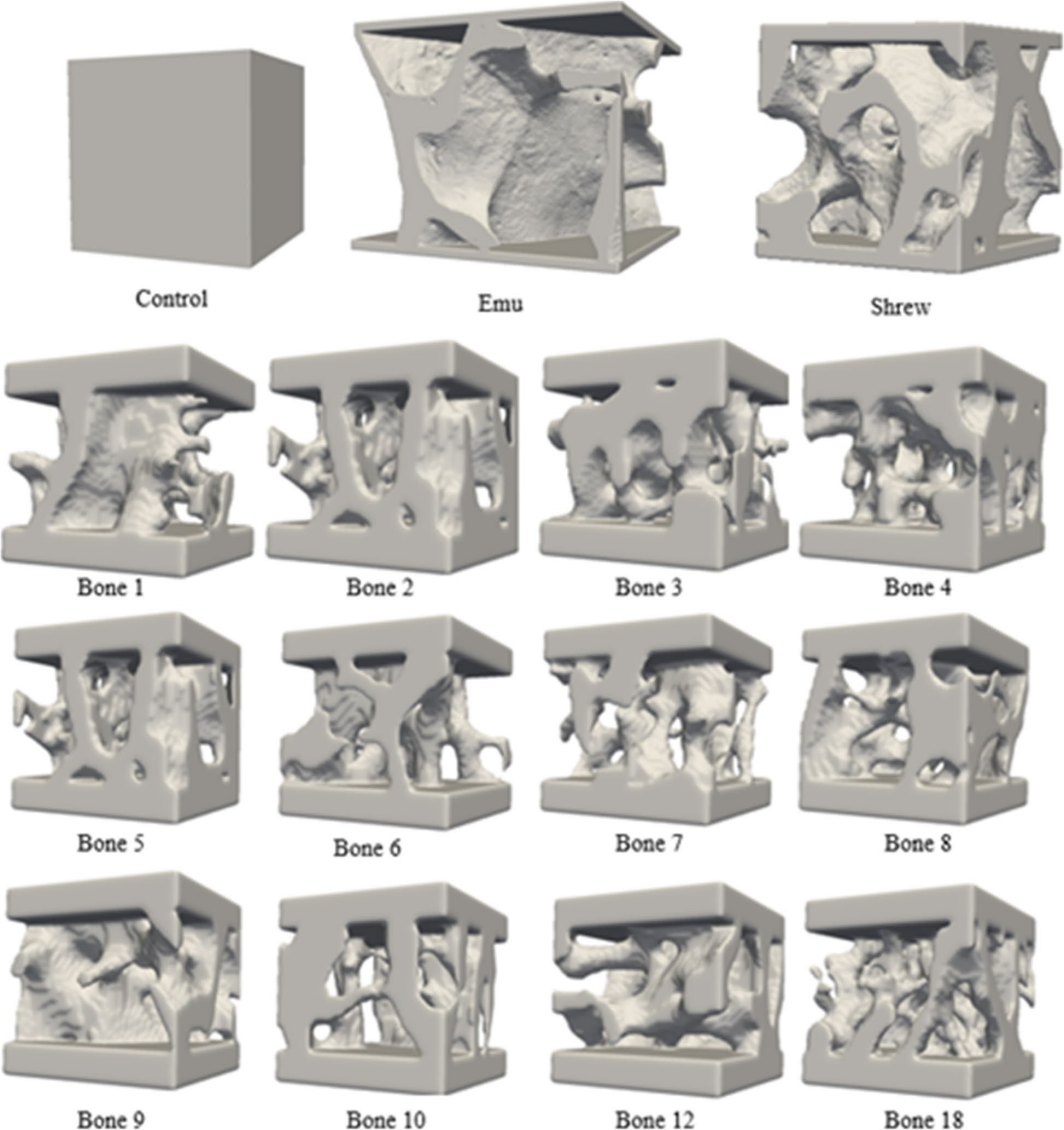
Overview of 3D printed phantoms visualized to scale. The control phantom was used to characterize the anisotropy and elastic modulus of the materials. The emu and shrew phantom were tested to build up to the complexity of the vertebral trabecular bone phantoms. Only 12 out of the 20 trabecular bone phantoms tested in this study are displayed

Platens were added to the top and bottom facets of the trabecular bone ROI to simulate compression plates in the z direction. Components unconnected to the main trabecular bone structure were removed to preserve only the largest connected components. The images were converted into surface meshes and then converted into tetrahedral meshes. The tetrahedral mesh was smoothed with a mutable diffusion Laplacian method to reduce noise, improve element shapes, and mesh quality [47]. Mesh simplification was performed by reducing the number of vertices and faces. Mesh smoothing and simplification allow for manageable mesh densities and prevent distortion in the shape of the triangles. The finalized meshes were saved as STL files to be 3D printed (Fig. 2).

The μFEA simulation used in this study models the linear elastic deformation of an object in uniaxial compression. In this simulation the boundary conditions are defined as a force applied in the axial (z) direction at the top facet vertices of the tetrahedral trabecular bone mesh, with the bottom facet vertices of the mesh remaining fixed. An additional constraint was imposed on the top facet vertices such that the vertices will only displace in the z direction, and not in the x and y directions. Finally, the material for each element is assumed to be isotropic and homogeneous, with a constant elastic modulus.

Input parameters defined for the simulation include elastic modulus and Poisson’s ratio. The specific value of the elastic modulus used in this simulation is determined by mechanical testing of a control phantom to reflect the true elastic modulus of the 3D printing resin. More details are provided in the subsequent section Material Characterization Test. A value of 0.3 was used for Poisson’s ratio after a sensitivity analysis showed that the stiffness value was insensitivity to this parameter. In the analysis, the Poisson’s ratio was varied from 0.10 to 0.45 to evaluate the effect on μFEA stiffness estimates. Modifying the Poisson’s ratio results in a stiffness variation of less than 5%. For Nylon 12 material, a Poisson’s ratio of 0.30 was determined to be acceptable [48].

The μFEA results return displacement, element centroids, von Mises stresses, element stresses, element strains, and total force for all the vertices and elements in the model. Stiffness values were calculated from the ratio of total force and vertical displacement of the top facet of the mesh. The simulation used the PyChrono simulation library and ParadisoMKL solver [49].

### Mesh convergence study

A study on mesh size was conducted to determine whether the mesh elements were sufficiently fine for μFEA stiffness results to converge. The number and size of mesh elements was varied for each individual tetrahedral mesh. As the number of mesh elements was decreased, the size of mesh elements increased. Finer tetrahedral meshes are expected to return more accurate results, however they require more computational resources [50]. The convergence study was run on all trabecular bone ROIs with equivalent boundary conditions and input parameters. The initial mesh was simplified by reducing the number of faces. A value denoted target fraction determines what fraction of the original number of faces the simplified mesh should contain. Given a mesh composed of 100 faces, a target fraction of 0.10 would yield 10 faces. The target fraction was varied from 0.015-0.60 to simplify each tetrahedral mesh from the initial size. The minimum mesh size for which the stiffness values converged was then determined. Stiffness values converge at approximately 30,147 mesh elements, further increasing the number of mesh elements resulted in less than 1% change in stiffness values.

### Additive manufacturing

We used Nylon 12 resin and EOS P396 with Process Software v3.7 Build 60 (Electro Optical Systems GmbH; Krailling, Germany) to print the phantoms. The STL files output by our μFEA model were imported into Magics 23.1 (Materialise; Leuven, Belgium). The meshes were visually inspected for abnormal features. No abnormal features or irregular geometry was detected in the surface meshes. Following visual inspection, all of the processed surface meshes were loaded in a simulated build plate in Magics. The trabecular bone phantoms were loaded parallel to the z axis of the 3D printing system. A powder cake is loaded into the tray of the SLS printer, lasers fusing the powder melting it iteratively until the finale print is complete. Once the phantoms were printed, they were removed from the powder cake and air blasted to remove excess powder.

Nylon 12 is known for its high resolution and versatility at rendering complex geometries [25]. The material properties of Nylon 12 resin and trabecular lumbar vertebrae are summarized in Table 1 [1, 24, 25, 51]. The following phantoms were printed using the SLS printer: control phantoms (50x50x50) mm, shrew trabecular bone phantoms (54x54x54) mm, emu trabecular bone phantoms (59x58x54) mm, and lumbar vertebrae trabecular bone phantoms (50x50x50) mm [41]. The control phantom is a solid block of resin that was printed to have a simple phantom that can be used to evaluate elastic modulus and anisotropy. The shrew and emu phantoms were selected as an additional validation of trabecular bone structures. Despite having simpler geometries, the animal bone ROIs have similar microstructure metrics to the lumbar vertebrae ROIs.

**Table 1:** Summary of mechanical properties of Nylon 12 resin and trabecular bone. The elastic modulus of trabecular bone is substantially higher than that of Nylon 12 resin

Rigid Materials	Tensile Strength (MPa)	Elongation at Break	Elastic Modulus (MPa)
Trabecular Bone	10-20	5-7%	14,800
Nylon 12	48	18%	1650

To assess the repeatability of additive manufacturing and mechanical testing, four ROIs were selected to be printed three times. The ROIs were uniquely identified as Bone 1, Bone 3, Bone 7, and Bone 18. These phantoms were then all mechanically tested. However, Bone 1 was excluded from μFEA analysis due to a mechanical testing error. The shrew and emu phantoms were also printed 3 times each to further assess repeatability.

### μCT of 3D printed phantoms

The trabecular bone phantoms were imaged with μCT to determine if the structures were faithfully reproduced during the 3D printing process, and to identify any 3D printing errors. Scanning with μCT is the gold standard for nondestructive characterization of porous 3D printed parts [52]. The phantoms were scanned with a 70 kVp X-ray source in air with a voxel size of 36.8 μm. The scans were analyzed to identify defects such as porosity, curling at the edge of the phantom, and unsintered powder. Printing errors also include structures fusing together or not being printed at all. Tree like porosities in the build direction can also be identified by μCT. These defects are a result of unconsolidated, unmelted, or unsintered powder particles.

### Image registration analysis

Image registration was used to assess the fidelity of the 3D printed phantom to the original ROI model. The ROI model was rasterized and the μCT image was resampled to match the dimensions and voxel size of the ROI. The rasterized ROI was labelled the fixed image, and the μCT image was labeled the moving image. A linear transformation was applied to the moving image, to align the trabecular structures with the fixed image. Once the proper alignment was achieved, a 3D rigid registration with six degrees of freedom was applied. The Dice score and Structural Similarity Index Measure (SSIM) were computed to evaluate the similarity between the fixed and moving images [53].

### Mechanical testing

3D printed phantoms were tested in compression (ASTM D695) with a 100 kN calibrated servohydraulic mechanics testing system (MTS) 312.21 frame, a 25 kN (204.51) servo hydraulic actuator, and a 15 kN 661.21A-04 load cell (MTS Systems Corporation, Eden Prairie, MN) [54]. Axial load and axial displacement data was collected from mechanical testing for all phantoms.

### Material characterization test

The control phantom was tested between two parallel platens and underwent uniaxial compression. To test for anisotropy, three control phantoms were tested perpendicular to the build direction, and three were tested parallel to the build direction. A small preload of approximately 50-300 N was applied with the top platen. Once the preload was applied, the top platen applied load at a constant rate until a maximum load of 14 kN was reached. The top platen returned to its original position of 1 mm after the maximum load was reached. Axial load and axial displacement data was collected and plotted. The mechanical stiffness of the phantom was calculated from the slope of the linear elastic region of the load displacement curve. The average mechanical stiffness was then used to compute the elastic modulus of the 3D printing resin (Eq. 1). This elastic modulus of 1461 MPa was used in the μFEA simulations to model the trabecular bone phantom compression.

1 $$Elastic\ Modulus=\frac{Stiffness\ \times Length\ of\ Object}{Cross\ Sectional\ Area}$$

### Mechanical testing trabecular bone phantoms

The trabecular bone phantoms underwent uniaxial compression testing between two parallel steel platens with a preload of 50-300 N. The load was applied at a constant velocity until reaching a maximum of 11kN. This load was chosen because it was close to the yielding point of the phantoms and provided a strong linear elastic response. The phantoms were not tested until fracture due to safety concerns. Once the maximum load was reached, the top platen relaxed at a constant velocity, and returned to the initial displacement of 1 mm.

**Fig. 3:**
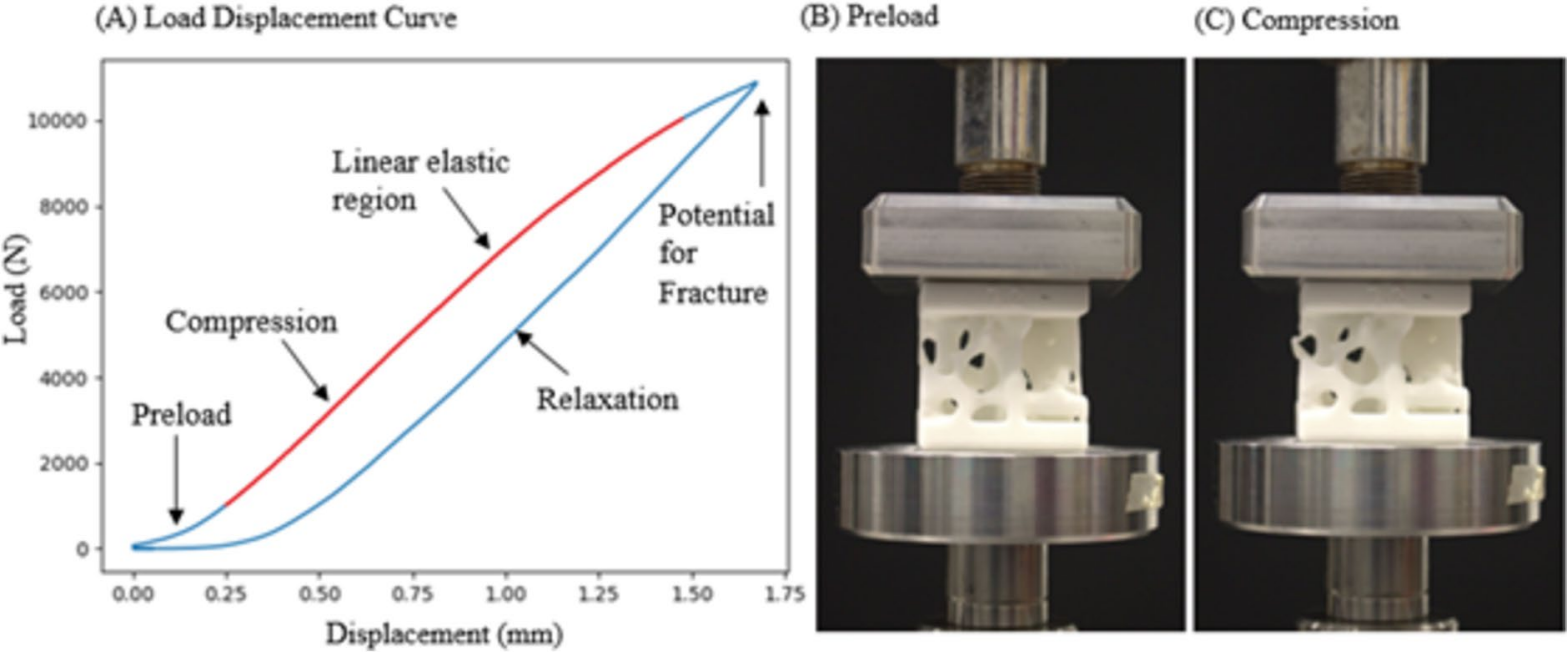
** A** Load-Displacement curve for linear compression test of trabecular bone phantom. The platen starts with an initial displacement of 1 mm above the phantom. Approximately 50-300 N of preload is first applied to the phantom, and then the phantom is compressed until a maximum load of 11 kN is reached. The phantom has the potential to fracture near 11 kN. Once this maximum load is reached, the platen relaxes and returns to its original displacement of 1 mm above the phantom. The linear elastic region of the load-displacement curve is isolated, which is marked in red. The slope of this region is used to estimate the mechanical stiffness of the phantom. **B** A bone phantom during preload. **C** The bone phantom during compression

A test was conducted to correct for the influence of the MTS frame on the phantom compression test results. The displacement results for the phantoms were overestimated which yielded an underestimate in stiffness values. To correct for this, a test was run with no phantom placed between the platens. The top platen was placed right above of the bottom platen with a preload of approximately 300 N. The platen then applied a constant load to the bottom platen until a load of 2.5 kN was reached. Once this load was reached, the top platen relaxed and returned to the original position. Axial load and axial displacement data were collected for this test. The frame’s load and displacement data were interpolated with mechanical testing results for the trabecular bone phantoms to calibrate phantom displacement. This returns a more accurate phantom displacement value and in turn a more accurate mechanical stiffness value. Load-displacement curves (Fig. 3) were plotted for all the tested trabecular bone phantoms and the slope of the linear elastic region was used to calculate mechanical stiffness.

## Results

### Microstructure metrics

There is a wide distribution of microstructure metrics across the trabecular bone phantoms, as indicated by the CoV, standard deviation, and range as displayed in Table 2. Phantoms range from healthy to osteoporotic. ROIs selected for 3D printing were chosen to have a range of structures that were realistic for healthy and osteoporotic bone. A diverse group of ROIs allows for a more robust evaluation of the agreement between mechanical testing and the μFEA simulations.

**Table 2:** Distribution of microstructure metrics for the trabecular bone samples. From the standard deviation, range and CoV it can be seen there is a distributive range in bone health. The dataset consists of bones ranging from healthy to osteoporotic

Parameter	Thickness(μm)	Spacing(μm)	Anisotropy	BV/TV	Connectivity ${\Big(}\frac{1}{\mu {m}^3}\Big)$	Ellipsoid Factor
Mean	365.15	1186.82	0.43	0.18	1.93 × 10^9^	0.11
Standard Deviation	51.9	185.60	0.11	0.05	1.75 × 10^9^	0.07
Min	298.91	912.50	0.25	0.08	4.70 × 10^8^	0.01
Max	537.19	1651.94	0.59	0.25	6.70 × 10^9^	0.25
CoV (%)	14	16	26	28	91	64

### Material characterization tests

The average mechanical stiffness of the perpendicular control phantom is 70,208.59 N/mm while the average mechanical stiffness of the parallel control phantom is 76,977.75 N/mm. The control phantoms printed with different orientations show there is an anisotropy in the Nylon 12 resin, as their stiffness results have a difference of 9.64%. There is an 11.39% difference between the elastic modulus computed from compression testing the control phantom (1461 MPa) and the manufacturer’s modulus (1650 MPa).

### Comparisons of mechanical and μFEA stiffness

**Fig. 4:**
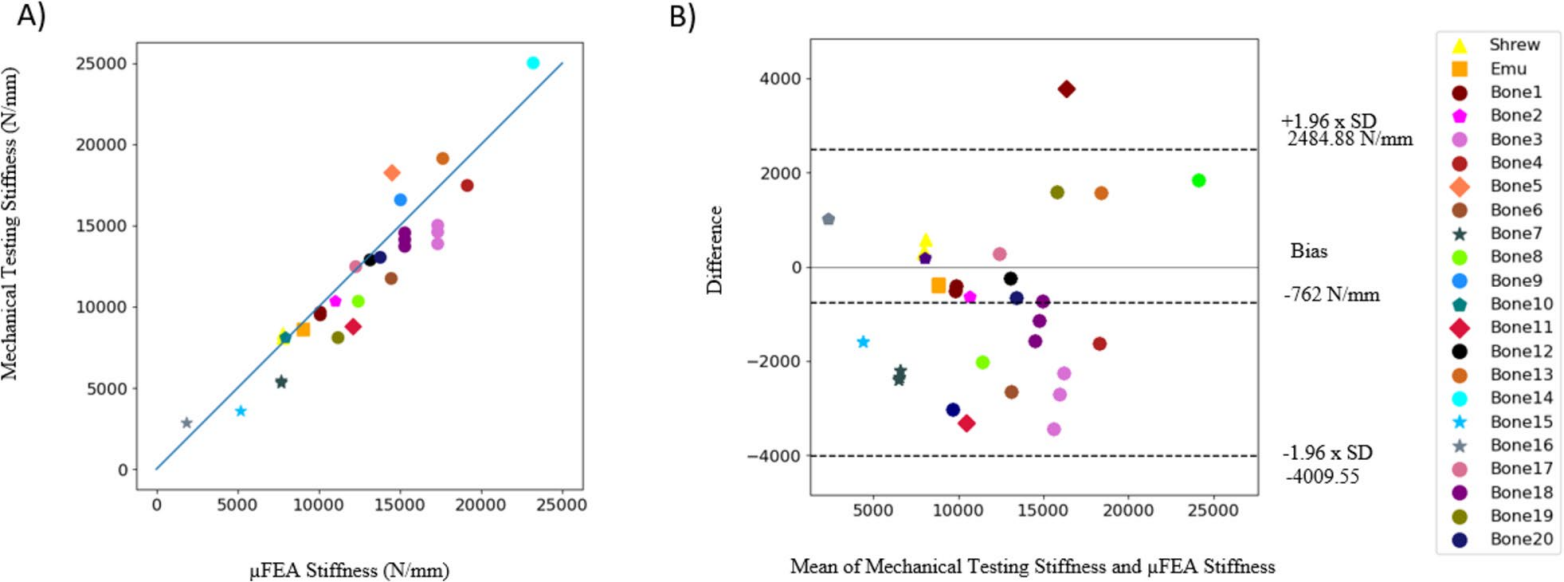
**A** Comparison between mechanical stiffness and μFEA estimated stiffness for all 3D printed phantoms. The overall high *r*^2^ =0.84 value indicates a good linear fit between mechanical and μFEA stiffness results. The Kendall correlation coefficient of 0.86 is indicative of correlation between mechanical and μFEA stiffness results. **B** The Bland-Altman analysis shows the agreement between mechanical and μFEA stiffness. The trabecular bone phantoms that fractured during mechanical testing are marked with a pentagon, bone phantoms that have high error are marked with a diamond, and bone phantoms that have both high error and fractured are marked with a star

Figure 4 plots the mechanical stiffness as a function of μFEA stiffness. These results indicate good agreement between mechanical and μFEA stiffness estimates (*R*^2^ of 0.84, RMSD of 1800.8 N/mm, NRMSD of 8.1%). The correlation between the mechanical stiffness and μFEA stiffness estimates is also strong, Kendall rank correlation coefficient of 0.86 and *p* < 0.05 [55]. This indicates a positive correlation between mechanical and μFEA stiffness. As the in silico phantom stiffness increases there is a comparative increase in the stiffness of the mechanically tested phantom.

Bland-Altman analysis assesses the limits of agreement between mechanical testing and μFEA analysis (Fig. 4). The results indicate there is agreement between the two methods. The two methods are well calibrated with mean stiffnesses for the mechanically tested phantoms and the μFEA simulations of 11,240.0 N/mm and 12,002.4 N/mm respectively. The limits of agreement are 2484.88 N/mm and − 4009.55 N/mm.

**Fig. 5:**
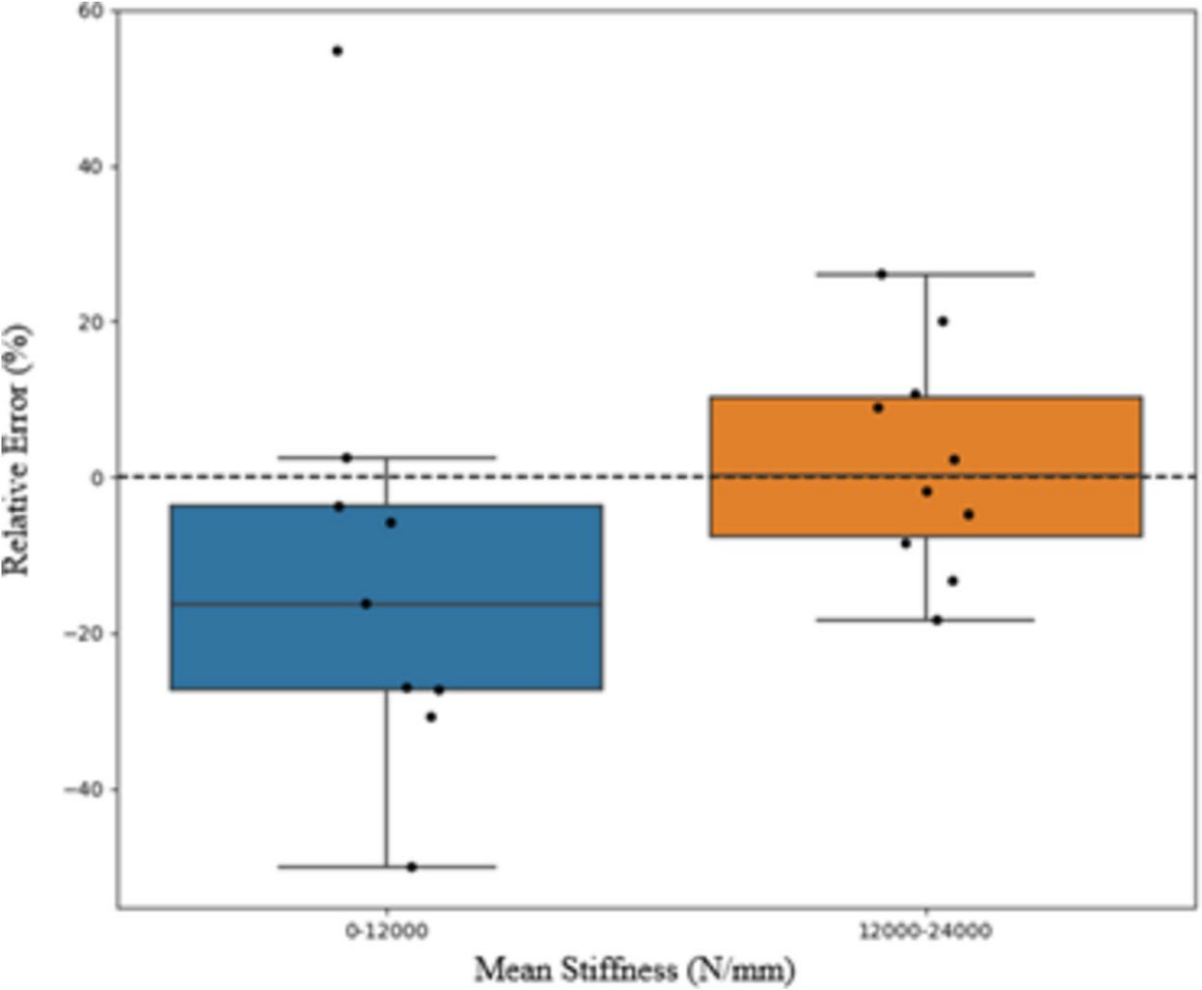
Relative error for all 20 unique trabecular bone phantoms, binned into low and high stiffness categories, below and above 12,000 N/mm, respectively. The x-axis is the mean of mechanical and μFEA stiffness of the trabecular bone phantoms, the y-axis shows the relative error between the mechanical and μFEA stiffness estimates. The error is largest for low stiffness bin compared with the high stiffness bin. The median error is represented in the plot by horizontal solid line in the box, − 11.6% median error for the low stiffness bin and 2.6% median error for the high stiffness bin. The low stiffness bin has a mean absolute error of 21.0% with the second bin having a mean absolute error of 8.5%

Relative error is more pronounced for phantoms with lower stiffness values. Figure 5 shows the relative error between mechanical and μFEA stiffness results for each trabecular bone phantom grouped by the mean stiffnesses that are above or below 12,000 N/mm. The average mechanical stiffness was used to represent phantoms with repeated prints. The error is larger in the range of 0-12,000 N/mm with mean absolute error of 21.0% in this range. In the range greater than 12,000 N/mm the mean absolute error is 8.5%. The median error of the two bins is − 11.6 and 2.6% respectively.

### Repeatability of mechanical testing

Table 3 provides the mechanically tested stiffness values for multiple repeated prints of the same object. The CoV was less than 5% for all the phantoms, with the shrew phantom having the lowest CoV at 0.2%. Overall, the mechanical stiffness results indicate high repeatability in our 3D printed phantoms as well as in mechanical testing.

**Table 3:** Repeatability assessment of mechanically derived stiffness results for 3D printed trabecular bone phantoms. The phantoms had a CoV of less than 5%, indicating the repeatability of mechanical testing and 3D printing is high

Volume	Number of Samples	Average (N/mm)	Standard Deviation (N/mm)	CoV (%)
Bone 3	3	14,515.83	595.58	4.1
Bone 7	3	5326.01	101.94	1.9
Bone 18	3	14,151.08	415.82	2.9
Shrew	3	8191.49	159.91	1.9
Emu	3	8636.25	20.84	0.2

### MicroCT of 3D printed phantoms

**Fig. 6:**
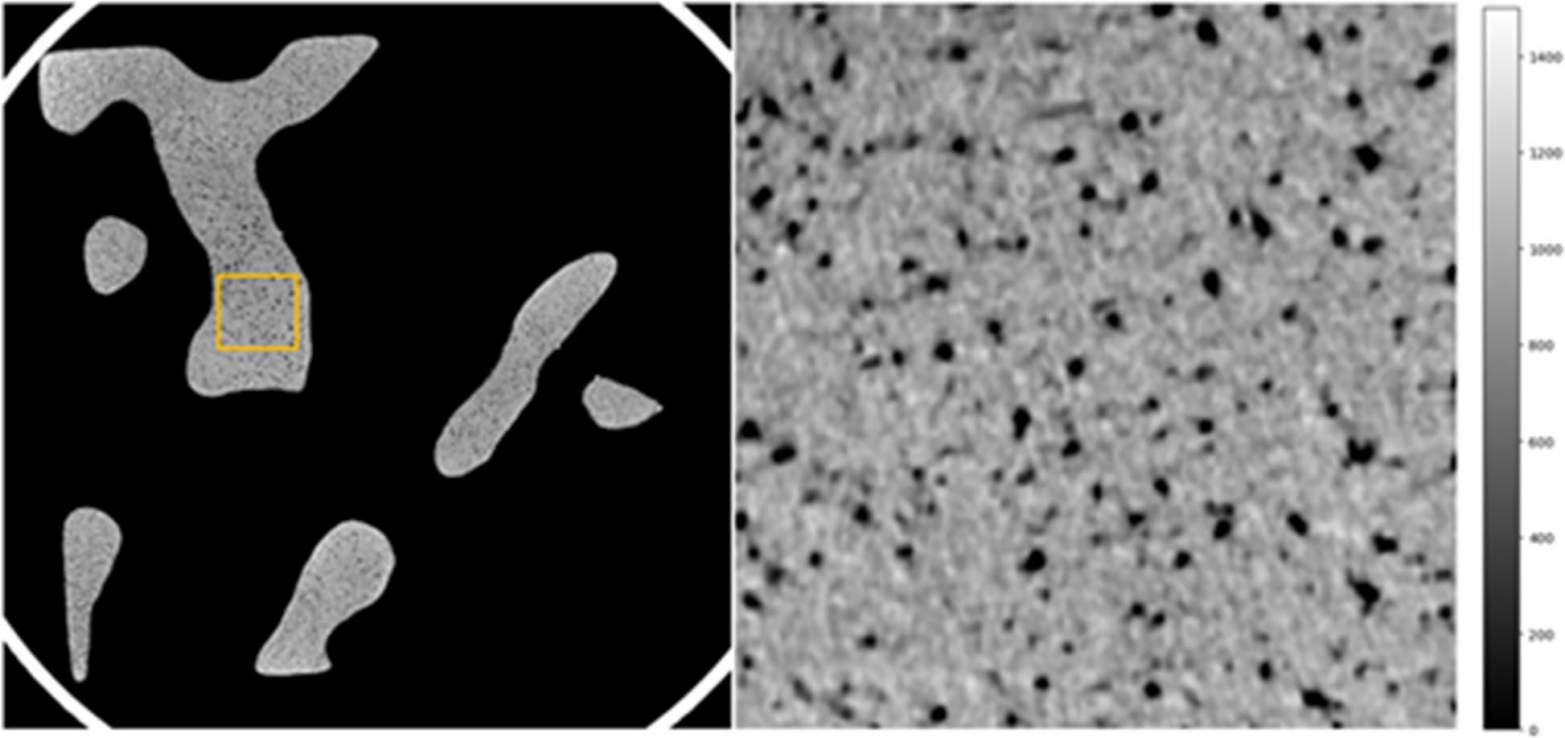
μCT scan of a 3D printed trabecular bone phantom showing pores, a 3D printing defect, within the trabecular structure

We show an example μCT image of a trabecular bone phantom in Fig. 6. This figure depicts 3D printing defects in the microstructure. Pores were distributed throughout the trabecular structures and were most prominent in the middle layers of the phantom. However, the porosity was not reflected in the in silico model. We did not observe other 3D printing defects such as substantial curling at the plates or unsintered powder.

### Image registration of trabecular bone phantoms

After image registration of the in silico bone model and the μCT image of the 3D printed phantoms, we estimated the average Dice score as 0.97 (95% CI, [0.96,0.98]) across all the trabecular bone phantoms.

**Fig. 7:**
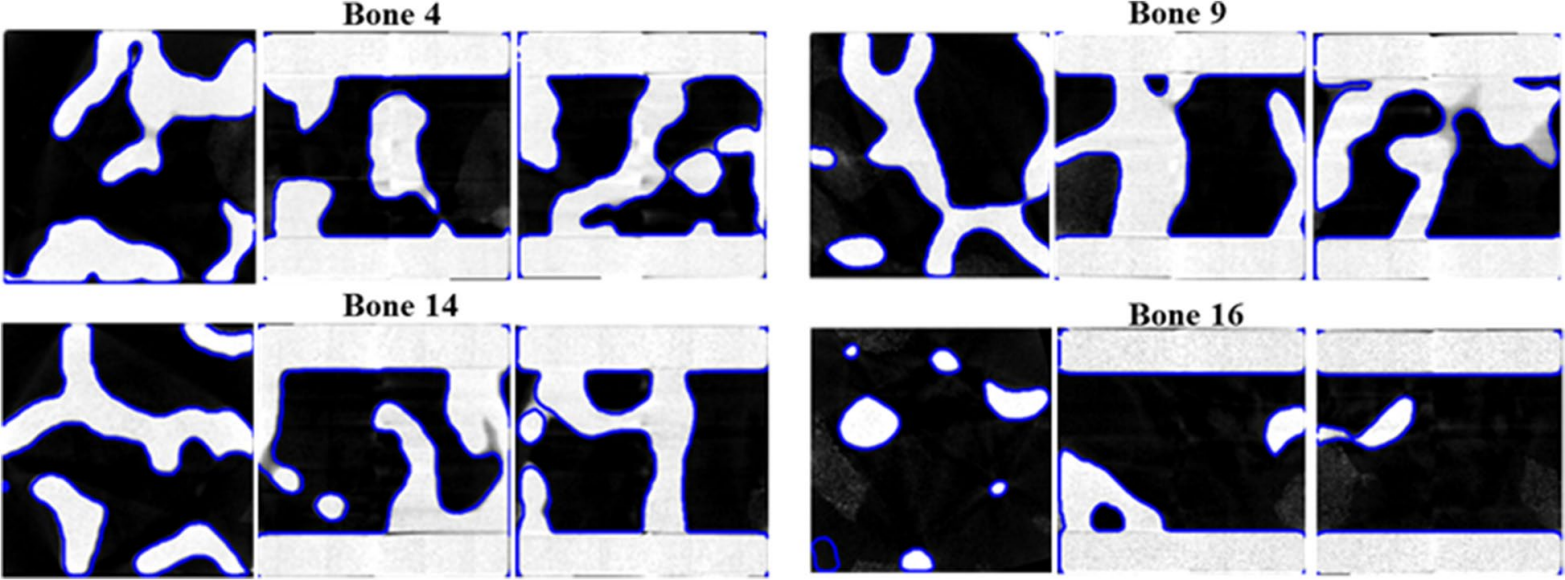
Representative triplanar overlays of in silico model of the ROI (blue outline) and the μCT images of the 3D printed trabecular bone phantom (grayscale image). Bone 4 and Bone 9 have high quality registrations achieving Dice scores greater than 0.97 and SSIM greater than 0.98. Bone 14 and Bone 16 have high Dice scores greater than 0.96 and SSIM greater than 0.97 but have registration errors due to image alignment issues. The overall high Dice score indicates that the 3D printing process was able to achieve high fidelity relative to the in silico ROI model

The Dice score ranged from 0.93-0.99 with a standard deviation of 0.022. We also assessed the average SSIM as 0.982 (95% CI, [0.981,0.983]) for the trabecular bone phantoms. The SSIM ranged from 0.978-0.0.986 with a standard deviation of 0.002. The Dice score and SSIM results indicate that the image registration accuracy is high, and that the 3D printed trabecular bone phantoms were generally produced with high fidelity. The SSIM results also indicate that additive manufacturing had minimal degradation on the overall shape of the ROI model. Figure 7 depicts examples of the original ROI models overlaying the μCT images of the phantom. Small registration errors were present in the z-axis plates and in the thin structures within the ROIs.

## Discussion

The μFEA stiffness estimates showed good agreement and correlation with mechanical testing results. Across all the trabecular bone phantoms the NRMSD was 8.1%, indicating an overall low error in μFEA stiffness estimates compared with the mechanical testing results. The NRMSD is lower than the observed change in bone stiffness as a result of the transition from healthy to osteoporotic bone, which is greater than 30% [56, 57]. Therefore, this is an acceptable amount of error between the model and mechanical testing. The repeatability of 3D printing and mechanical testing is high, as the CoV is less than 5% for mechanically derived stiffness values. Both μFEA and mechanical testing may be applicable to evaluating bone stiffness for assessment of bone health.

There are several errors that can contribute to the discrepancy between mechanical stiffness and μFEA stiffness. One source of error is the mechanical testing boundary conditions. Phantoms may have been placed slightly off axis from the platens yielding inaccurate stiffness measurements. A misalignment of 20° can cause a decrease in 40% of the measured elastic modulus [51]. In this study it is likely that phantoms were placed off axis by 1-2°. Misalignment can result in load being applied nonuniformly to the phantom. This can lead to additional displacement in the x and y directions, as observed in Bone 7. There are also some discrepancies in the μFEA simulation and our mechanical testing setup. The boundary conditions for the μFEA only simulates force in the z direction on the phantom with no displacement in the x and y directions. Thus, Bone 7 would be expected to have a large error between mechanical and μFEA stiffness because of the displacement in the x and y directions. Overall, phantoms with low stiffness values are more sensitive to the effects of off axis loading which may be why we observed larger errors for phantoms with stiffnesses below 12,000 N/mm than for the higher stiffness phantoms.

Further discrepancy between the μFEA and mechanical stiffness values can be explained by evaluating the 3D printing quality. The μCT image registration analysis showed that the overall structures were printed with high fidelity. Registration errors were observed in some trabecular structures. These errors were considered minimal and restricted to only a few phantoms. 3D printing defects such as unsintered powder, curling, and deformation at the edge of the plates were not observed in our printed phantoms. However, this does not mean the resin microstructure of the ROI model is correctly represented in the phantoms. Despite high fidelity in 3D printing of the overall shape, material properties such as anisotropy and porosity may still have biased the mechanical testing results.

We observed porosity in the μCT scans of the 3D printed phantoms. The top and bottom layers of the phantom have the lowest degree of porosity, while the middle layers containing the trabecular structures were the most porous. Porosity has a negative effect on a material’s structural integrity as pores introduce variations in the mechanical properties [31]. The specific arrangement of pores across layers of the phantom can also negatively influence mechanical properties. Additionally, pores aligned transversal to the phantom’s build direction will introduce anisotropy [30]. The phantom porosity may contribute to the 11.39% difference between the elastic modulus computed from the control phantom and the manufacturer’s modulus.

An additional source of error is the inherent anisotropy of the 3D printing resin [30]. Control phantom testing results show there is at least a 9.64% difference in stiffness results when the phantom is printed orthogonal or in parallel to the build direction. As trabecular bone structures are in a variety of orientations, this anisotropy in the 3D printing resin can aggregate and become a source of error in the 3D printed phantom. The anisotropy is not reflected in the in silico model and is an additional source of error between the 3D printed phantom and the model. The inherent anisotropy of the resin can also contribute to the difference between the experimental elastic modulus and the manufacturer’s modulus. However, an in-depth quantitative analysis of the impact of this anisotropy on the material is outside the scope of the current work.

There are several limitations in the study design. It is difficult to 3D print phantoms with many trabecular structures with high fidelity. The phantoms printed with the SLS printer are anisotropic and porous causing discrepancies between simulated and experimental results. This anisotropy can be difficult to model due to the various orientations of trabecular bone structures. Additionally, this work only used one type of 3D printer for generating phantoms. Thus, it is unknown if results from a different printer would have better or worse agreement with μFEA stiffness estimates. Furthermore, it is challenging to 3D print phantoms at native resolution, thus we must determine scaling for the trabecular bone phantoms. Further limitations include only mechanical testing and modeling of the linear elastic region. While the linear elastic region was able to provide good agreement between the mechanical and μFEA stiffness results, there may be nonlinear behavior present during compression of the trabecular bone phantoms that is not simulated by the μFEA. As a result, inaccurate stiffness values would be returned by μFEA. Lastly, it is unclear whether mechanical testing error, nonlinear behavior, 3D printing defects, or the material properties had the largest influence on the error present in the study.

Despite these limitations a major advantage of this study was that we printed a diverse set of trabecular bone ROIs extracted from several lumbar vertebrae. As a result, we were able to test a variety of cases to thoroughly evaluate the agreement between mechanical testing and μFEA simulations.

The results of this study have identified a 3D printing method and scaling at which real trabecular bone structures can be reliably produced. Since the phantom has both consistent morphology and mechanics, a calibration can be applied to account for differences in the elastic modulus as well as scaling. The μFEA simulation can potentially be applied to real trabecular bone and show good agreement with in vitro mechanical testing. To find good agreement for real trabecular bone a few assumptions must be made. First, the elastic modulus of real trabecular bone can be assumed to be homogenous throughout the sample [58], as was also assumed for the trabecular bone phantoms. Secondly, in this study, we were able to find good agreement between mechanical testing and μFEA as the phantoms were an accurate reproduction of the trabecular bone microarchitecture. For real trabecular bone, the surface mesh must accurately reflect the true microarchitecture of the samples. The bones will need to be imaged with sufficient spatial resolution and corrected for partial volume artifacts. Given these two conditions are met, the μFEA model is expected to show good agreement with mechanical testing results of real trabecular bone.

## Conclusion

In this work, we developed a diverse set of in silico 3D trabecular bone structures. The structures were printed as physical phantoms using an SLS printer. The agreement between stiffness values derived from μFEA simulation of the in silico models and mechanical testing of the 3D printed phantoms was evaluated. We demonstrated that the stiffness values derived from the two methods agree to within 8.1% across the trabecular bone phantoms. Discrepancies between the mechanical stiffness and μFEA stiffness estimations can be attributed to mechanical testing errors, 3D printing defects, resin anisotropy, and errors in μFEA modeling. Our findings suggests that despite the potential sources of error in our measurements, the agreement between the two measurements support additive manufacturing as a method for validating μFEA stiffness predictions. Overall, the level of agreement achieved between the mechanical stiffness and μFEA indicates that our μFEA methods may be acceptable for assessing bone mechanics of complex trabecular structures as part of an analysis of overall bone health. However, while this work is a first step to evaluating trabecular bone stiffness using 3D printing, to evaluate overall fracture risk in patients a model of the entire vertebral body is required, not just an ROI [59]. The entire vertebral body would have all relevant bone structures including cavities and poor trabeculae that are important contributors to fracture risk.

## Abbreviations

μFEA: Micro-Finite Element Analysis
μCT: Micro-computed tomography
DEXA: Dual Energy X-ray Absorptiometry
BMD: Bone mineral density
MDCT: Multidetector computed tomography
HR-pQCT: High-resolution peripheral quantitative computed tomography
ROIs: Regions of interests
SLS: Selective laser sintering
SLA: Stereolithography
ASTM: American Society for Testing and Materials
BV/TV: Bone volume fraction
EF: Ellipsoid factor
RMSD: Root mean square deviation
NRMSD: Normalized root mean square deviation
CoV: Coefficient of variation
SSIM: Structural Similarity Index Measure
